# Antibodies Against the NH_2_-Terminus of the GluA Subunits Affect the AMPA-Evoked Releasing Activity: The Role of Complement

**DOI:** 10.3389/fimmu.2021.586521

**Published:** 2021-02-26

**Authors:** Francesca Cisani, Guendalina Olivero, Cesare Usai, Gilles Van Camp, Stefania Maccari, Sara Morley-Fletcher, Anna Maria Pittaluga

**Affiliations:** ^1^ Pharmacology and Toxicology Section, Department of Pharmacy, DIFAR, Genoa, Italy; ^2^ Institute of Biophysics, National Research Council, Genoa, Italy; ^3^ Univ. Lille, CNRS, UMR 8576 - UGSF - Unité de Glycobiologie Structurale et Fonctionnelle, Lille, France; ^4^ International Associated Laboratory (LIA), “Prenatal Stress and Neurodegenerative Diseases”, University of Lille – CNRS, UGSF UMR 8576/Sapienza University of Rome and IRCCS Neuromed, Lille, France; ^5^ Department of Science and Medical - Surgical Biotechnology, University Sapienza of Rome, Rome, Italy; ^6^ IRCCS San Martino Hospital, Genova, Italy

**Keywords:** synaptosomes, AMPA receptors, GluA2 and GluA3 subunits receptor, glutamate exocytosis, complement, C1q complement, cortex, autoimmune diseases

## Abstract

Antibodies recognizing the amino-terminal domain of receptor subunit proteins modify the receptor efficiency to controlling transmitter release in isolated nerve endings (e.g., synaptosomes) indirectly confirming their presence in these particles but also allowing to speculate on their subunit composition. Western blot analysis and confocal microscopy unveiled the presence of the GluA1, GluA2, GluA3, and GluA4 receptor subunits in cortical synaptosomes. Functional studies confirmed the presence of presynaptic release-regulating AMPA autoreceptors in these terminals, whose activation releases [^3^H]D-aspartate ([^3^H]D-Asp, here used as a marker of glutamate) in a NBQX-dependent manner. The AMPA autoreceptors traffic in a constitutive manner, since entrapping synaptosomes with the pep2-SVKI peptide (which interferes with the GluA2-GRIP1/PICK1 interaction) amplified the AMPA-evoked releasing activity, while the inactive pep2-SVKE peptide was devoid of activity. Incubation of synaptosomes with antibodies recognizing the NH_2_ terminus of the GluA2 and the GluA3 subunits increased, although to a different extent, the GluA2 and 3 densities in synaptosomal membranes, also amplifying the AMPA-evoked glutamate release in a NBQX-dependent fashion. We then analyzed the releasing activity of complement (1:300) from both treated and untreated synaptosomes and found that the complement-induced overflow occurred in a DL-t-BOA-sensitive, NBQX-insensitive fashion. We hypothesized that anti-GluA/GluA complexes in neuronal membranes could trigger the classic pathway of activation of the complement, modifying its releasing activity. Accordingly, the complement-evoked release of [^3^H]D-Asp from antiGluA2 and anti-GluA3 antibody treated synaptosomes was significantly increased when compared to untreated terminals and facilitation was prevented by omitting the C1q component of the immunocomplex. Antibodies recognizing the NH2 terminus of the GluA1 or the GluA4 subunits failed to affect both the AMPA and the complement-evoked tritium overflow. Our results suggest the presence of GluA2/GluA3-containing release-regulating AMPA autoreceptors in cortical synaptosomes. Incubation of synaptosomes with commercial anti-GluA2 or anti-GluA3 antibodies amplifies the AMPA-evoked exocytosis of glutamate through a complement-independent pathway, involving an excessive insertion of AMPA autoreceptors in plasma membranes but also affects the complement-dependent releasing activity, by promoting the classic pathway of activation of the immunocomplex. Both events could be relevant to the development of autoimmune diseases typified by an overproduction of anti-GluA subunits.

## Introduction

Over the past 20 years, several studies endorsed the hypothesis that inflammatory processes involving components of innate immunity play important roles in the pathophysiology of central neurological disorders (1–3). The hypothesis originated from the identification of antigen-specific CNS immune responses in a rare group of cancer-triggered disorders, e.g., the paraneoplastic syndrome (4–6). It was demonstrated that autoimmune-mediated responses against neuronal proteins causes severe forms of encephalitis often associated with epileptic symptoms (7–9). The tumorigenic nature of the disease, however, was not a prerequisite, since an anomalous production of autoantibodies recognizing accessible epitopes of central self-proteins was also detected in patients suffering from non-infectious encephalitis that were not associated to tumors.

Today there is a large consensus that an aberrant activation of autoimmunity processes represents a recurrent sign of immune pathologies typified by neuropsychiatric symptoms (amnesia, seizures, psychosis). Furthermore, the seroprevalence of autoantibodies targeting ion-channels or neuronal receptors including NMDA receptors, α-amino-3-hydroxy-5-methyl-4-isoxazolepropionic acid (AMPA) receptors, GABA_A_ receptors, and dopamine D2 receptors in the cerebral spinal fluid of patients suffering from central disorders supports the main role of these autoimmune components in the diseases progression (7, 10). Although attractive, the hypothesis needs to be definitively proven, e.g., by highlighting a clear causative link connecting the pathological production of autoantibodies and the molecular/cellular events underlying the neurological symptoms (11–13).

An emerging hypothesis considers that autoantibodies recognizing the extracellular domains of membrane receptors are pathogenic because of their ability to interfere with the respective targets at the neuro-glial surface. Particularly, it is proposed that autoantibodies raise against the N-terminal domain sequence of receptor/transporter proteins can influence the insertion, localization, and function of the respective antigens. It is the case of the autoantibodies recognizing the NMDA receptor subunits (e.g., the GluN1 and the GluN2A and B subunits) (14–16) which interfere with the functions of NMDA receptors. Interestingly, comparable results were obtained also when studying the impact of the serum of patients enriched with anti-GluN antibodies. Both the commercial antibodies and the patient’s sera containing auto anti-GluN auto-antibodies accelerate the internalization of the NMDA receptors in neurons, impairing their signaling at chemical synapses (17, 18).

Differently from the anti-GluNs, the impact of autoantibodies targeting the subunits involved in the expression of the AMPA receptors (the GluA1 to 4 proteins) is matter of debate. Contrasting results emerged when studying the impact of anti-GluA autoantibodies from patients suffering immuno-encephalitis and those elicited by commercial anti-GluA antibodies (19). The contrasting findings also might dependent on the different cascade of events triggered by the anti-GluA auto-antibodies, which also were reported to involve a complement-mediated pathways (20, 21).

Functional adaptations induced by antibodies recognizing the N-terminal of membrane receptors subunits also could be detected in isolated nerve endings (we refer to as synaptosomes). In particular, commercially available antibodies recognizing the NH2-sequence of receptor subunits affect the receptor-mediated control of transmitter exocytosis from these particles and can be used as pharmacological tools for the characterization of the subunit composition of metabotropic (22–25) and ionotropic (26) receptors in synaptosomes isolated from different CNS regions (“the immunopharmacological approach”) (23, 26).

We recently demonstrated that antisera of patients suffering from frontotemporal dementia enriched in anti-GluA3 antibodies impeded the releasing activity of presynaptic release-regulating AMPA autoreceptors in cortical synaptosomes (27). Based on these observations, we turned to commercially available anti-GluA antibodies to verify their impact on the functional activity of presynaptic release-regulating AMPA receptors in cortical glutamatergic nerve endings and possibly to predict the subunit composition of the receptor under study. We also investigated whether the presence of the antibody-antigen complex in synaptosomal plasma membranes could activate complement-evoked responses then influencing its releasing activity in these terminals. The results unveiled a complex scenario involving a *complement-independent* and a *complement-dependent* cascade of events that could be relevant to improve the knowledge of the pathological mechanisms occurring in the cortex of patients with circulating anti-GluA antibodies.

## Materials and Methods

### Animals

Mice (male, strain C57BL/6J) were purchased from Charles River (Calco, Italy) and housed in the animal facility of DIFAR, Section of Pharmacology and Toxicology, in environmentally controlled conditions (ambient temperature = 22°C, humidity = 40%) and under a regular 12-h light/dark cycle with food and water *ad libitum*. Mice were euthanized by cervical dislocation, followed by decapitation, and the cortex rapidly removed. The experimental procedures were in accordance with the European legislation (European Communities Council Directive of 24 November 1986, 86/609/EEC) and the ARRIVE guidelines, and they were approved by the Local Committee for Animal care and welfare of the University of Genova and the Italian Ministry of Health (DDL 26/2014 and previous legislation; protocol number n° 75F11.N.IMY). Experiments were performed following the Guidelines for Animal Care and Use of the National Institutes of Health and in accordance with the Society’s Policies on the Use of Animals and Humans in Neuroscience Research. In line with the 3Rs rules (replacement, refinement, and reduction), any effort was made to reduce the number of animals to obtain statistically reliable results.

### Isolation of Synaptosomes

Synaptosomes are resealed round particles having a diameter of about 1 µm that originate from nerve terminals (28). These particles are acutely isolated from fresh-tissue and then separated and purified with gradient centrifugation. Synaptosomes retain the functional and the structural features of the nerve terminals they originate from, confirming their presynaptic origin. They contain mitochondria, vesicles for the storage of transmitter, cytosolic structures, and part of the endoplasmic reticulum and are specialized for transmitter release and second messenger productions. Furthermore, they express enzymes and transporters accounting for the uptake, the storage, and the release of transmitters. These events are physiologically controlled by naïve receptors located presynaptically on synaptosomal membranes, the activation of which control the functional activities of the isolated nerve endings, including the release of transmitter. In few cases, synaptosomes retain fragments of the postsynaptic membranes that are attached to the presynaptic membranes by means of proteins (including PSD95 and ephrin) that are specialized in bridging the pre to the post synaptic component of the synaptic active zone. The fragments of the postsynaptic membranes usually do not re-seal and cannot influence the presynaptic-induced effects.

Cortical purified synaptosomes were prepared by homogenizing the cortical tissue with a glass/Teflon tissue grinder (clearance 0.25 mm) in 10 volumes of 0.32 M sucrose, buffered to pH 7.4 with Tris-(hydroxymethyl)-amino methane (TRIS, final concentration 0.01 M). The homogenate was centrifuged at 4°C at 1,000 × g for 5 min to remove nuclei and debris, and the resulting supernatant was gently stratified on a discontinuous Percoll® gradient (6%, 10%, and 20% v/v in Tris-buffered sucrose) and then centrifuged at 33,500 × g for 5 min. The synaptosomal fraction (the layer between 10% and 20% Percoll®) was subsequently collected and washed by centrifugation with a physiological solution having the following composition: NaCl, 140 mM; KCl, 3 mM; MgSO_4_, 1.2 mM; CaCl_2_, 1.2 mM; NaH_2_PO_4_, 1.2 mM; NaHCO_3_, 5 mM; HEPES, 10 mM; glucose, 10 mM; pH 7.2–7.4). When indicated, cortical tissue was homogenized in buffered sucrose containing 20 µM of the peptides under investigation (e.g., pep2-SVKI and pep2-SVKE) in order to entrap these agents into subsequently isolated synaptosomes (see 29, 30).

### Release Study

Release studies were carried out by applying a technique first proposed by Maurizio Raiteri and colleagues in 1974 that is called “the up-down superfusion of a thin layer of synaptosomes” and that is widely recognized as an approach of choice to study the transmitter release and its regulation by presynaptic receptors (31, 32). The major advantage of the experimental approach is that any substance released by synaptosomes is immediately removed by the up-down superfusing solutions, so that its concentration at nerve terminals is minimized. Thus, the continuous superfusion avoids indirect effects due to endogenous substances, since compounds released by one particle cannot act on adjacent isolated terminals. Moreover, the geometry of the system and the selective labelling of terminals with radioactive tracers (or the measurement of endogenous transmitters) impede artefacts that may originate from the presence of different families of terminals.

Cortical synaptosomes were incubated at 37°C in a rotary water bath for 30 min in the absence (control) or presence of the following antibodies recognizing the NH_2_ terminus of the GluA proteins (final concentration 1:500): rabbit polyclonal anti-GluA1; rabbit monoclonal anti-GluA2; mouse monoclonal anti-GluA3; rabbit monoclonal anti-GluA4. At t = 15 min of incubation, the radioactive tracer [^3^H]D-aspartate ([^3^H]D-Asp, f.c.: 50 nM) was added until the end (t = 30 min). Identical portions of the synaptosomal suspensions were then layered on microporous filters at the bottom of parallel thermostated chambers in a Superfusion System (Ugo Basile, Comerio, Varese, Italy) (31, 33). Stratified synaptosomes were superfused at 0.5 ml/min with the standard physiological solution for 36 min to equilibrate the system. At t = 36 min four 3-min superfusate fractions (i.e., namely: b1, from 36 to 39 min; b2, from 39 to 42 min; b3 from 43 to 45min, and b4 from 45 to 48) were collected.

When indicated, at t = 39 min of superfusion, synaptosomes were exposed to (S)AMPA (in the presence of 10 µM cyclothiazide) till the end of the superfusion period (t = 48 min). In the experiments dedicated to studying the impact of the complement and C1q-depleted complement, these immune components were added at the end of the first fraction collected for 90 s, then replaced with the superfusion medium. When indicated, the AMPA antagonist NBQX and the excitatory amino acid transporter blocker DL-t-BOA were added concomitantly to the agonists.

The amount of radioactivity released into each superfusate fraction was expressed as a percentage of the total synaptosomal tritium content (fractional efflux). For each experimental condition, tritium release was calculated as the sum of the tritium (expressed as %) in the four fractions collected. The agonist-evoked overflow was calculated as the difference between the % of tritium released from synaptosomes exposed in superfusion to (S)AMPA or to complement and that from synaptosomes superfused with the physiological medium.

In all the figures, data are reported as the mean ± SEM of independent determinations obtained in different experiments run in triplicate (three superfusion chambers for each experimental condition).

### Confocal Microscopy

Mouse cortical synaptosomes (40 μg of protein) were fixed with 2% paraformaldehyde, permeabilized with 0.05% Triton X-100 phosphate-buffered saline (PBS), and incubated overnight at 4°C with the following primary antibodies diluted in 3% albumin PBS: rabbit polyclonal anti-GluA1 (1:1000), rabbit monoclonal anti-GluA2 (1:200), mouse monoclonal anti-GluA3 (1:500), rabbit monoclonal anti-GluA4 (1:500), goat polyclonal anti-syntaxin 1A (1:4000), and guinea pig anti-vesicular glutamate transporter type 1 (VGLUT1; 1:1000). Incubated synaptosomes were then washed in PBS and incubated for 1 h at room temperature with the respective secondary antibodies: donkey anti-rabbit AlexaFluor-647 and donkey anti-goat AlexaFluor-488 or goat anti-guinea pig AlexaFluor-488 (1:1000, colocalization of GluA1**/**GluA2**/**GluA3 receptor proteins with syntaxin 1A or VGLUT1), and, donkey anti-mouse AlexaFluor-647 and donkey anti-goat AlexaFluor-488 or goat anti-guinea pig AlexaFluor-488 (1:1000, colocalization of GluA3 receptor proteins and syntaxin 1A or VGLUT1). Synaptosomes were then applied onto coverslips (34). Fluorescence imaging (512 × 512 × 8 bit) acquisition was performed by a six-channel Leica TCS SP5 laser-scanning confocal microscope, equipped with 458, 476, 488, 514, 543, and 633 nm excitation lines, through a plan-apochromatic oil immersion objective 63X/1.4NA. Light collection configuration was optimized according to the combination of chosen fluorochromes. Sequential channel acquisition was performed to avoid spectral bleed-through artifacts. Leica **‘**LAS AF**’** software package was used for image acquisition, storage and visualization. The quantitative estimation of co-localized proteins was performed as already described (26, 35), by calculating the “co-localization coefficients” (36) (WCIF Colocalization Plugins, Wright Cell Imaging Facility, Toronto Western Research Institute, Canada) in the Image J 1.51w software (Wayne Rasband, NIH, USA).

### Western Blot Analysis

Mouse cortical purified synaptosomes were lysed in modified RIPA buffer (10 mM Tris, pH 7.4, 150 mM NaCl, 1 mM EDTA, 0.1% SDS, 1% Triton X-100, 1mM sodium orthovanadate and protease inhibitors) and quantified for protein content with BCA assay. Samples were boiled for 5 min at 95°C in SDS-PAGE sample buffer. Proteins were separated on 10% precast polyacrylamide gel (Bio-Rad) by means of SDS–polyacrylamide gel electrophoresis and then blotted onto PVDF membrane. Membranes were blocked for 1 h at room temperature with Tris-buffered saline-Tween (t-TBS: 20 mM Tris, pH 7.4, 150 mM NaCl, and 0.05% Tween 20) containing 5% (w/v) non-fat dried milk, and then probed with the following primary antibodies overnight at 4°C: rabbit polyclonal anti-GluA1 (1:1000), rabbit monoclonal anti-GluA2 (1:2000), mouse monoclonal anti-GluA3 (1:500), rabbit monoclonal anti-GluA4 (1:500). After three 5-min washes in t-TBS, membranes were incubated for 1 h at room temperature with the appropriate horseradish peroxidase-linked secondary antibodies. After three 10-min washes in t-TBS protein bands were detected with an ECL (enhanced chemiluminescence) western blotting detection system. Images were acquired using the Alliance LD6 images capture system (Uvitec, Cambridge, UK) and analyzed with UVI-1D software (Uvitec, Cambridge, UK).

### Biotinylation Studies

Changes in the cortical synaptosomal surface levels of GluA2 and GluA3 receptor proteins were evaluated by biotinylation and immunoblot analyses (37). Briefly, mouse cortical synaptosomes were divided into four aliquots. One aliquot was lysed in modified RIPA buffer to analyze the GluA subunit content in the total synaptosomal lysate (L). The remaining three aliquots were incubated for 30 min at 37°C under mild shaking in the absence (control synaptosomes) or presence of rabbit anti-GluA2 antibody (1:500, anti-GluA2 incubated synaptosomes) and of mouse anti-GluA3 antibody (1:500, anti-GluA3 incubated synaptosomes). The control (C) and the anti-GluA incubated synaptosomes were then labelled for 1 h at 4°C with sulfo-NHS-SS-biotin (1.5 mg/ml) in a medium with the following composition (mM): 138 NaCl, 2.7 KCl, 1.8 KH_2_PO_4_, 10 Na_2_HPO_4_, 1.5 MgCl_2_, 0.2 CaCl_2_, pH 7.4 (PBS/Ca-Mg). The biotinylation reaction was then stopped by incubating synaptosomes with PBS/Ca-Mg containing 100 mM glycine for 20 min at 4°C. After two washes, synaptosomes were lysed in modified RIPA buffer and identical samples (100 µg) incubated with Dynabeads MyOne Streptavidin T1 beads for 30 min at room temperature under shaking to pull-down the biotinylated proteins. Dynabeads were also added to the non-biotinylated synaptosomes to check the specificity of streptavidin pull-down (B). After extensive washes, all the samples were boiled for 5 min at 95°C in SDS-PAGE loading buffer to isolate biotinylated proteins from the beads. Eluted fractions were analyzed through immunoblot assay (see Immunoblotting analysis section). The immunoreactivity of GluA2 and GluA3 receptor proteins was monitored by using rabbit anti-GluA2 (1:2000) and mouse anti-GluA3 (1:500) antibodies in the total lysate (L), in control (C), in anti-GluA2 or anti-GluA3 incubated biotinylated synaptosomes (anti-GluA2 and anti-GluA3 incubated synaptosomes) as well as in the streptavidin pull-down of the non-biotinylated synaptosomal lysate (B).

### Statistics and Calculations

Sigma plot 10 data analysis and graphing software package was used for data handling/statistics and for graph drawing. Analysis of variance was performed by ANOVA followed by Dunnett’s multiple-comparisons test, as appropriate; direct comparisons were performed by Student’s *t*-test. The level of significance was set at p < 0.05.

### Chemicals

[2,3-^3^H]D-aspartate (specific activity 11.3 Ci/mmol) was from Perkin Elmer (Boston, MA, USA). (S)AMPA, pep2-SVKI, pep2-SVKE, 2,3-Dioxo-6-nitro-1,2,3,4-tetrahydrobenzo[f]quinoxaline-7-sulfonamide disodium salt (NBQX), and DL-threo-β-Benzyloxyaspartic acid (DL-t-BOA) were purchased by Tocris Bioscience (Bristol, UK). Horseradish peroxidase-conjugated anti-mouse and anti-rabbit secondary antibodies and glycine were from Sigma (Milan, Italy). Luminata Forte Western blotting detection system, guinea pig anti-vesicular glutamate transporters type 1 (VGLUT1) antibody and mouse anti-GluA3 monoclonal antibody (MAB5416) were purchased from Millipore (Temecula, CA, USA). Rabbit anti-GluA1 polyclonal antibody (ab86141) and rabbit anti-GluA2 monoclonal antibody (ab206293, EPR18115) were from Abcam (Cambridge, UK). Rabbit anti-GluA4 monoclonal antibody was from GeneTex (GTX62957, Irvine, USA). Donkey anti-rabbit AlexaFluor-647, goat anti guinea pig AlexaFluor-688, donkey anti-mouse AlexaFluor-647, and donkey anti-goat AlexaFluor-688 were from Life Technologies Corporation (Carlsbad, CA, USA). Anti-syntaxin 1A monoclonal mouse IgG, was purchased from Chemicon (CA;USA). Complement and C1q-depleted complement was from Gentaur (Kampenhout, Belgium).

## Results

### The Glutamatergic Synaptosomes Isolated From the Cortex of Adult Mice Are Endowed With the GluA Subunits

**Figure 1:**
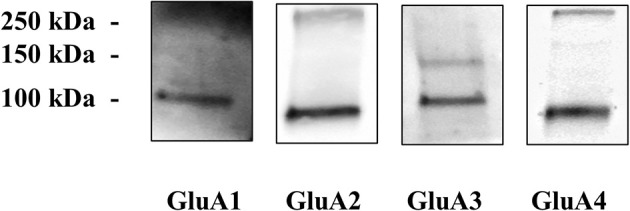
Cortical synaptosomal plasma membranes are endowed with GluA1 to 4 receptor subunits. Western blot analysis was carried out in mouse cortical lysates to evidentiate the presence of GluA1, GluA2, GluA3, and GluA4 receptor proteins (see *Materials and Methods* for technical aspects). The figure shows a representative blot of four analyses carried out on different days.

First, the presence of AMPA receptors in cortical nerve endings was assessed by evaluating the existence of the GluA1 to 4 subunit proteins in synaptosomal plasma membranes isolated from cortical synaptosomes. The Western blot analysis of the cortical synaptosomal lysates unveiled the presence of the four GluA subunits (**Figure 1**). Each antibody recognized a component with a mass consistent with the monomeric form of the respective receptor subunit (~110 kDa) (38–40).

**Figure 2:**
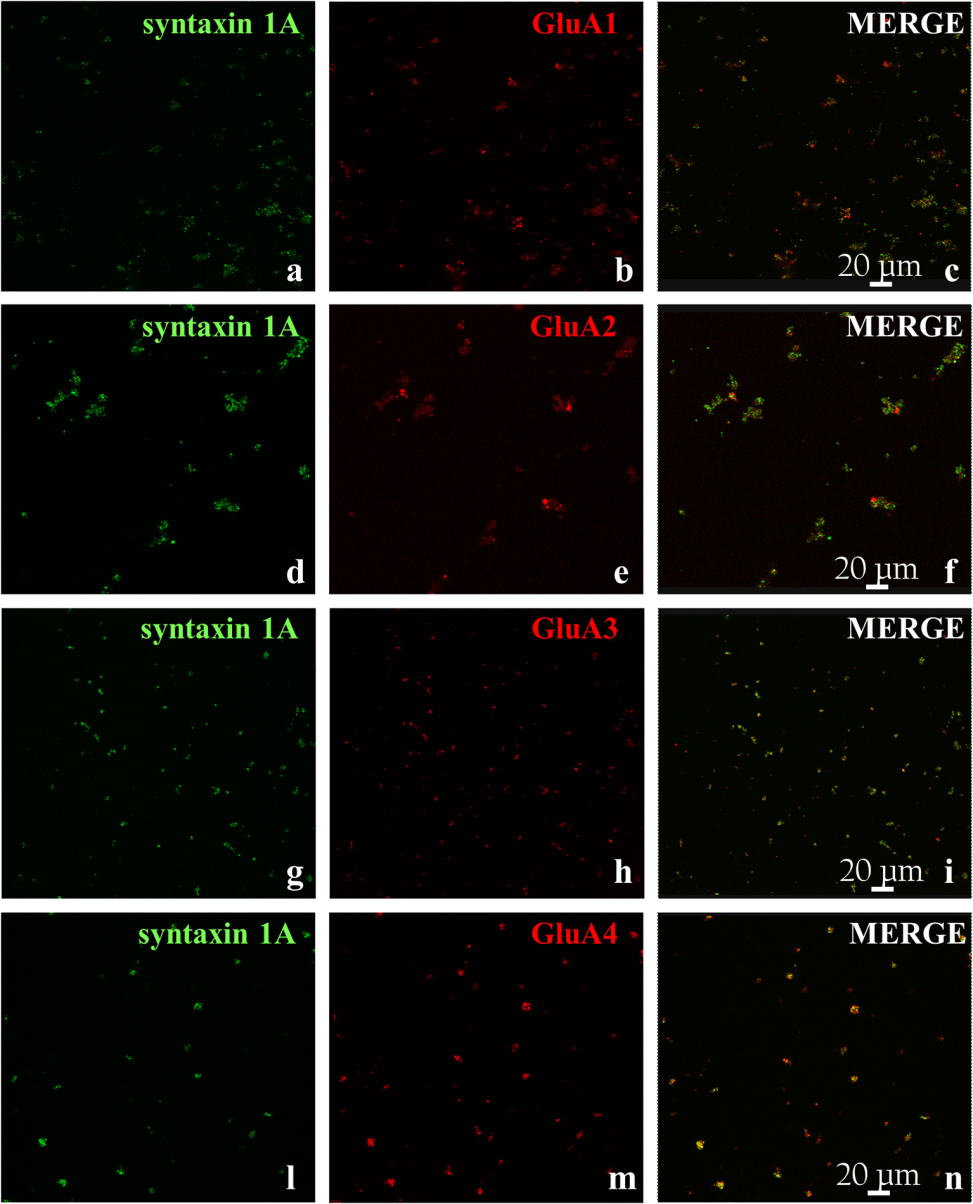
GluA receptor subunits colocalize with syntaxin 1A in mouse cortical synaptosomes. Confocal analysis of the GluA1 (red, **B**), GluA2 (red, **E**), GluA3 (red, **H**), and GluA4 (red, **M**) subunit immunoreactivities in syntaxin 1A-positive cortical nerve terminals (green, **A**, **D**, **G**, **L**, respectively) and their colocalization (merge, yellow, **C**, **F**, **I**, **N**, respectively). The figure shows representative images of three independent experiments.

Then, we verified the presence of the AMPA receptors in cortical synaptosomes with confocal microscopy, by analyzing the presence of the GluA1 to 4 subunits in syntaxin 1A-positive particles. The representative images in **Figure 2** clearly show that the GluA 1, 3, and 4 subunits are expressed in cortical syntaxin 1A-immunopositive particles to comparable levels (e.g., GluA1: 44 ± 5 %; GluA3: 46 ± 9 % and GluA4: 41± 11 %, results expressed as percentage of the syntaxin 1A-immunopositive terminals, respectively), while the GluA2 subunit is express to a low level (27 ± 5% of the syntaxin 1A-immunopositive synaptosomes).

**Figure 3:**
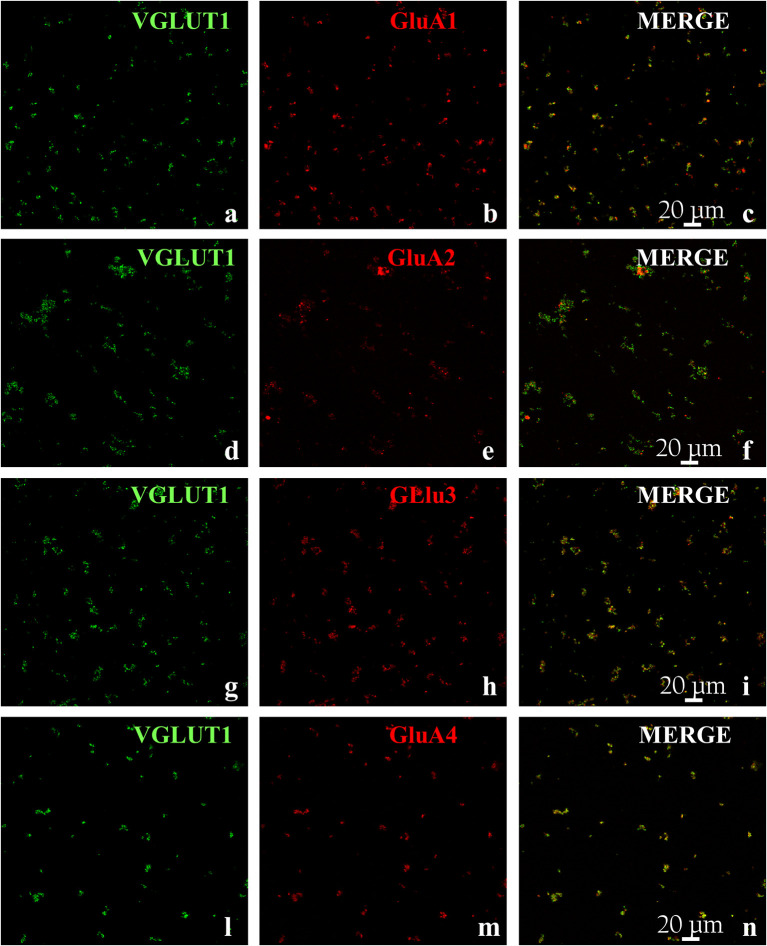
Colocalization of GluA subunits with the Vesicular Glutamate Transporter type 1 in mouse cortical synaptosomes. Confocal analysis of the GluA1 (red, **B**), GluA2 (red, **E**), GluA3 (red, **H**), and GluA4 (red, **M**) subunit immunoreactivities in VGLUT1-positive cortical nerve terminals (green, **A**, **D**, **G**, **L**, respectively) and their colocalization (merge, yellow, **C**, **F**, **I**, **N**, respectively). The figure shows representative images of three independent experiments.

It is known that cortical synaptosomes are an heterogenous ensemble of nerve endings originating from different neuronal subpopulations. In an attempt to study whether and how the GluA subunits exist in glutamatergic cortical nerve endings, the glutamatergic synaptosomal subpopulation was identified as VGLUT1 positive structure (**Figures 3A, D, G, L**) and analyzed for its immunopositivity for the anti-GluA1 (**Figure 3B**), the anti-GluA2 (**Figure 3E**), the anti-GluA3 (**Figure 3H**) and the anti-GluA4 (**Figure 3M**) antibodies, respectively. The analysis of the anti-VGLUT1- positive terminals unveiled that a large percentage of the glutamatergic synaptosomes also express the GluA proteins (**Figures 3C, F, I, N**; GluA1 = 91±3%; GluA2 = 83±3%; GluA3 = 92±4%; GluA4 = 77±4%, results expressed as percentage of the VGLUT1-immunopositive terminals, respectively). These observations confirmed the presence of the GluA receptor subunits in cortical glutamatergic synaptosomes.

### The Anti-GluA Antibodies Differentially Affect the (S)AMPA-Evoked [^3^H]D-Aspartate Release

**Figure 4:**
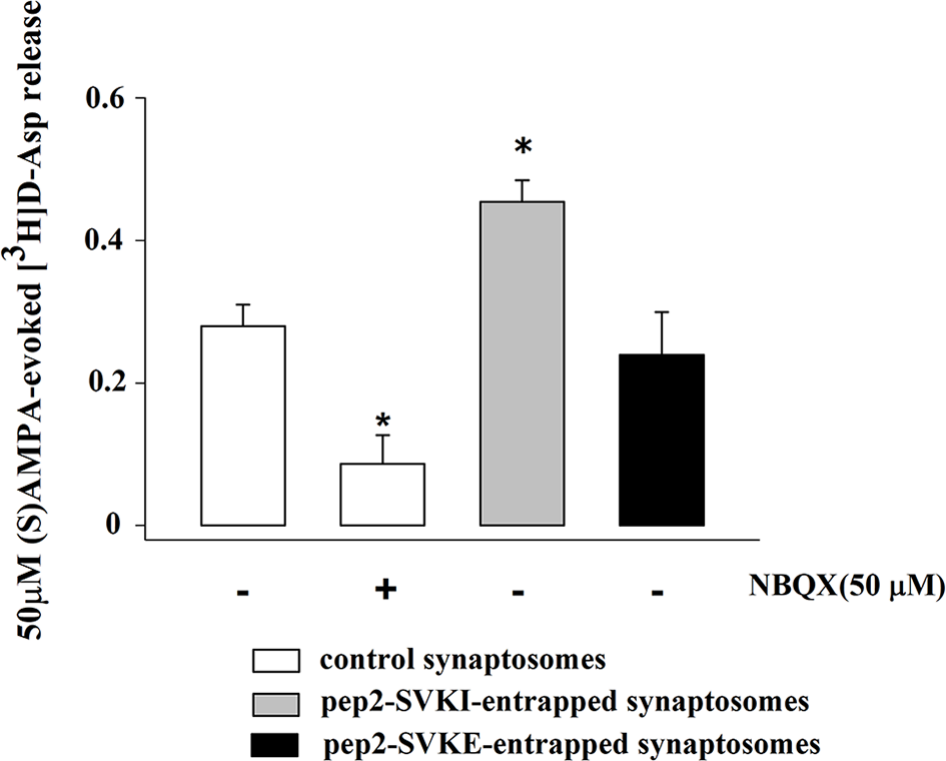
(S)AMPA evokes the release of [^3^H]D-aspartate from mouse cortical synaptosomes: antagonism by NBQX and effects of peptides that interfere with AMPA receptor trafficking. Synaptosomes were preloaded with the radioactive tracer and exposed in superfusion to 50 µM (S)AMPA in the presence of 10 µM cyclothiazide to monitor tritium exocytosis. NBQX (50 µM) was added concomitantly to the agonists. Peptides were entrapped into synaptosomes as reported in the Method section. The (S)AMPA-evoked overflow was calculated as release above the spontaneous release and it amounted to 0.32 ± 0.02 (% of the total synaptosomal tritium content). Results are expressed as agonist-evoked tritium overflow. Results are expressed as mean ± SEM of data from at least three experiments run in triplicate (three superfusion chambers for each experimental condition). *p < 0.05 versus 50 µM (S)AMPA.

Experiments were then carried to verify the functional consequences of the activation of presynaptic release-regulating AMPA autoreceptors in the cortical synaptosomes. To this aim, we quantified the release of glutamate by applying the “up-down superfusion of a thin layer of synaptosomes”, an experimental approach first proposed by Raiteri and colleagues in 1974 (31), that is widely recognized as appropriate to study the mechanism of release of transmitter and their regulation by presynaptic auto and hetero receptors (see also 32, 41, 42). Cortical synaptosomes were labelled with [^3^H]D-Asp, a non-metabolizable glutamate analogue routinely used as a marker for the endogenous aminoacid in release studies (23, 26), and exposed in superfusion to 50 µM (S)AMPA to quantify whether and how the spontaneous release of the radioactive tracer is modified following exposure of synaptosomes to the agonist. A significant increase in the release of tritium was detected that was largely prevented by the concomitant addition of the AMPA antagonist NBQX [50 µM; **Figure 4** further confirming that activation of presynaptic release-regulating AMPA autoreceptors accounted for the releasing effect, but see (43)].

Synaptosomes were then incubated with the anti-GluA antibodies applied in confocal microscopy and western blot analyses to confirm with the “immunopharmacological approach” (22–26) the presence of the presynaptic release regulating AMPA autoreceptors and possibly to hypothesize their subunit composition.

**Figure 5:**
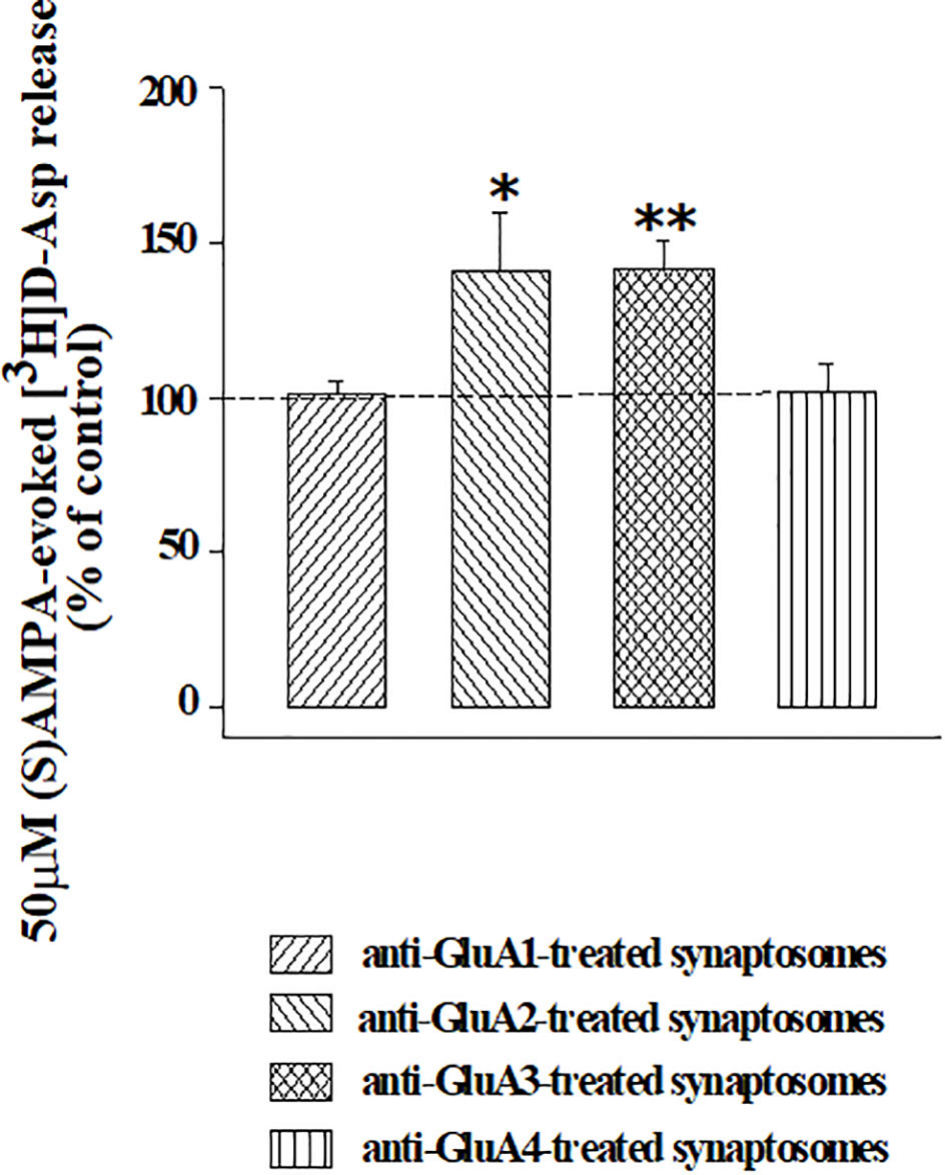
Effects of the incubation of cortical synaptosomes with anti-GluA receptor antibodies on the (S)AMPA-evoked release of [^3^H]D-Aspartate. Mouse cortical synaptosomes were incubated in the absence (control) or in the presence of the anti-GluA1 (rising right hatched bar), of the anti-GluA2 (rising left hatched bar), of the anti-GluA3 (crossed hatched bar) or of the anti-GluA4 (vertical hatched bar) antibody and the release of preloaded [^3^H]D-Asp was elicited by exposing synaptosomes in superfusion the (S)AMPA (50 µM) in the presence of cyclothiazide (10 µM). Results are expressed as percentage of the (S)AMPA-evoked release from control synaptosomes (% of control) and are reported as mean ± SEM from at least three experiments run in triplicate. *p < 0.05 versus control; **p < 0.01 versus control.

The releasing activity elicited by (S)AMPA (50 µM) was largely conserved in synaptosomes that were treated with anti-GluA1 or anti-Glu4 antibodies when compared to that observed in control synaptosomes (e.g., synaptosomes that were not incubated with an antibody), but significantly increased in isolated nerve endings that were incubated with the anti-GluA2 or the anti-GluA3 antibodies (**Figure 5**). The incubation of synaptosomes with each of the four anti-GluA antibodies did not affect the basal release of [^3^H]D-Asp (**Table 1**), consistent with the conclusion that facilitation of the (S)AMPA-evoked release of tritium was accountable for by antibody-induced adaptation of the presynaptic release-regulating AMPA receptors. Accordingly, the (S)AMPA-evoked release of tritium from anti-GluA2 or anti-GluA3-treated antibodies was largely prevented by 50 µM NBQX concomitantly added to the agonist (anti-GluA2 incubated synaptosomes : 0.06 ± 0.02 % of tritium release over basal release, corresponding to the 18.33 % of residual of the (S)AMPA-evoked [^3^H]D-Asp release; anti-GluA3 incubated synaptosomes : 0.07 ± 0.04 % of tritium release over basal release, corresponding to the 21.38 % of residual of the (S)AMPA-evoked [^3^H]D-Asp release; results are expressed as (S)AMPA-evoked [^3^H]D-Asp, n = 3 experiments, *p < 0.05 versus respective control), to a level comparable to that observed in control synaptosomes.

**Table 1:** The incubation of synaptosomes with the anti-GluA antibodies fails to affect the basal release of preloaded [^3^H]D-aspartate.

	[^3^H]D-aspartate release (% of the synaptosomal content)
Control synaptosomes	0.63 ± 0.05, (n=7)
Anti-GluA1-incubated synaptosomes	0.70 ± 0.06, (n=5), *n.s.*
Anti-GluA2-incubated synaptosomes	0.61 ± 0.02, (n=5), *n.s.*
Anti-GluA3-incubated synaptosomes	0.76 ± 0.07, (n=5), *n.s.*
Anti-GluA4-incubated synaptosomes	0.75 ± 0.06, (n=5), *n.s.*

Identical aliquots of mouse cortical synaptosomes were incubated with the anti-GluA antibodies and the release of preloaded [^3^H]D-aspartate in the first fraction collected (the basal release) quantified and expressed as percentage of the total [^3^H]D-aspartate synaptosomal content. The data are reported as mean ± SEM of n (value within brackets) experiments run in triplicate (three superfusion chambers for each experimental condition); n.s. not significative.

### The Incubation of Synaptosomes With Anti-GluA2 or AntiGluA3 Antibodies Modify the Density of GluA2 and the GluA3 Receptor Subunits in Plasma Membranes

**Figure 6:**
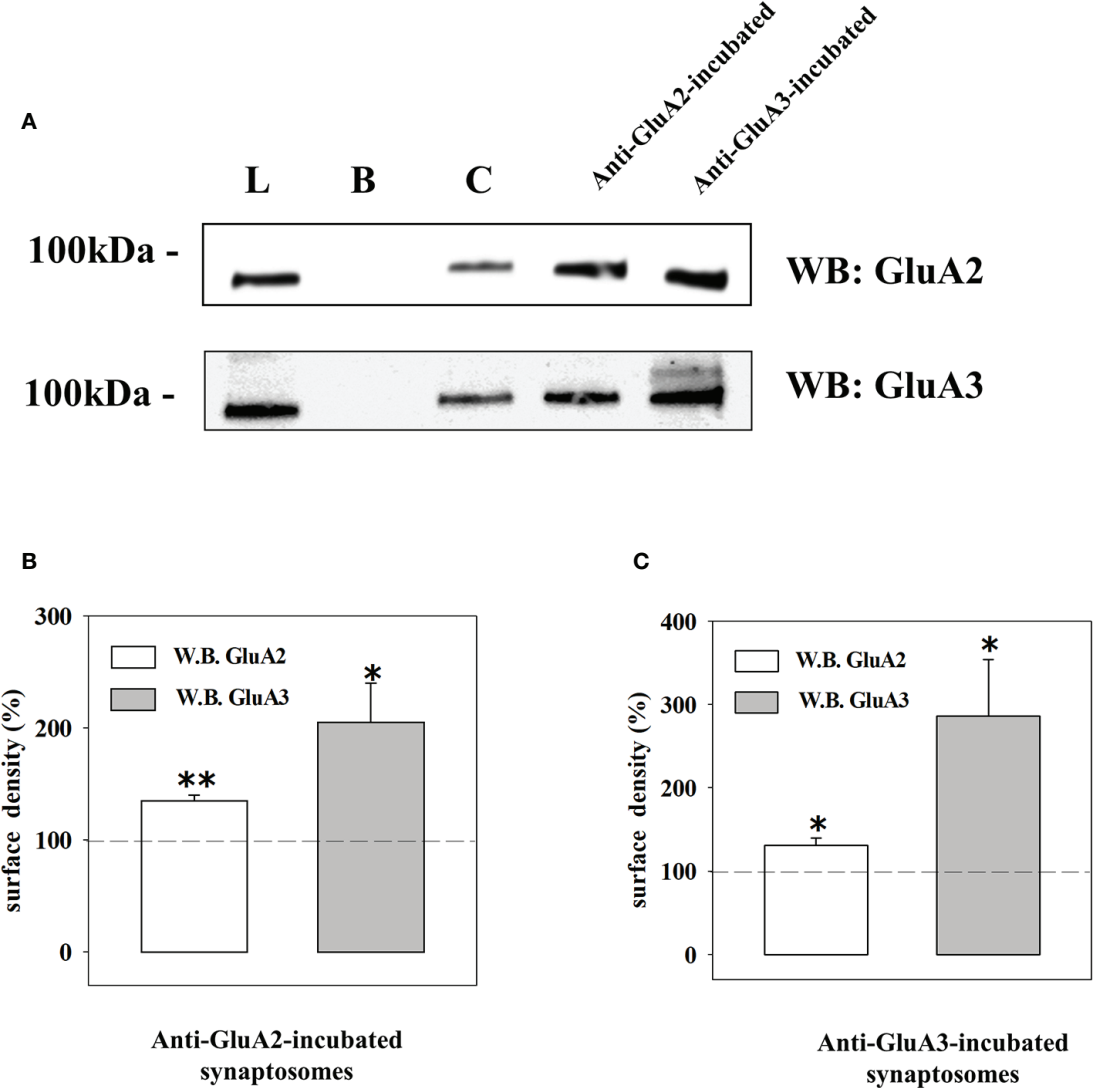
Western blot analysis of the GluA2 and GluA3 subunits surface densities in cortical terminals that were incubated with the anti-GluA2 and the anti-GluA3 antibodies. **(A)** Western blot analysis of the GluA2 (upper) and of the GluA3 (lower) immunoreactivity in the total synaptosomal lysate (L), the biotin-untreated synaptosomal lysate **(B)**, the control biotin-treated synaptosomal lysate **(C)**, the lysates from biotin-treated synaptosomes incubated with the anti-GluA2 and the anti-GluA3. The blot is representative of three different experiments runs on different days. Changes in GluA2 (white bar) and in GluA3 (grey bar) subunits surface density in anti-GluA2 **(B)** or anti-GluA3 **(C)** incubated synaptosomes. The results are expressed as percentage of the respective control and are reported as mean ± SEM. *p < 0.05 versus respective control; **p < 0.01 versus respective control.

The quantification of the density of the biotin-tagged GluA2 and GluA3 subunit proteins in control synaptosomes (i.e., synaptosomes that were not incubated with an antibody) and in synaptosomes that were incubated with the anti-GluA2 antibody (**Figures 6A, B**) or the anti-GluA3 antibody (**Figures 6A, C**) unveiled changes in the density for the respective receptorsubunits in the synaptosomal plasma membranes. Particularly, incubation with the anti-GluA2 antibody caused significant increase in the GluA2 density, but hugely affected the GluA3 component. Similarly, incubation of synaptosomes with the anti-GluA3 antibody caused a dramatic increase in the insertion of the GluA3 subunit in synaptosomal plasma membranes and a low, significant increment in GluA2 density. Indirectly, these results could suggest that the AMPA autoreceptors controlling glutamate release from cortical nerve endings could traffic in-out the synaptosomal plasma membranes. It is known that AMPA receptors are dynamically distributed between the cytosolic an the membrane compartment (44, 45) and that in synaptosomes they move in-out the synaptosomal plasma membranes in a constitutive manner (30). The constitutive trafficking involves the internalization of AMPA receptors mediated by the interaction between the carboxy tail of the GluA2 subunit and the glutamate receptor-interacting protein 1(GRIP1) or the protein interacting with C kinase 1 (PICK1) (46). This interaction can be disrupted by enriching synaptosomes with pep2-SVKI, a peptide corresponding to the last 10 aminoacid sequence of the intracellular C-terminal domain of the GluR2 subunit that associates with the PDZ motifs of GRIP1 and PICK1. Once in the cytosol, pep2-SVKI competes with the GluA2 C-terminal tail for binding to the scaffolding proteins, then preventing receptor internalization. This event emerges as an increase of the (S)AMPA-evoked releasing activity due to the increased number of AMPA receptors in synaptosomal plasma membranes (30, 39). The effect of pep2-SVKI was compared to that of pep2-SVKE, an inactive peptide usually used as a negative control. Entrapping either the pep2-SVKI or the pep2-SVKE peptide within cortical synaptosomes failed to affect the spontaneous release of [^3^H]D-Asp (not shown) but significantly increased the (S)AMPA-evoked release of tritium (**Figure 4**), suggesting that the AMPA autoreceptor under study moves in-out synaptosomal plasma membranes in a constitutive manner.

### The Incubation of Synaptosomes With Anti-GluA Antibodies Differently Affects the Complement-Evoked Glutamate Overflow

**Figure 7:**
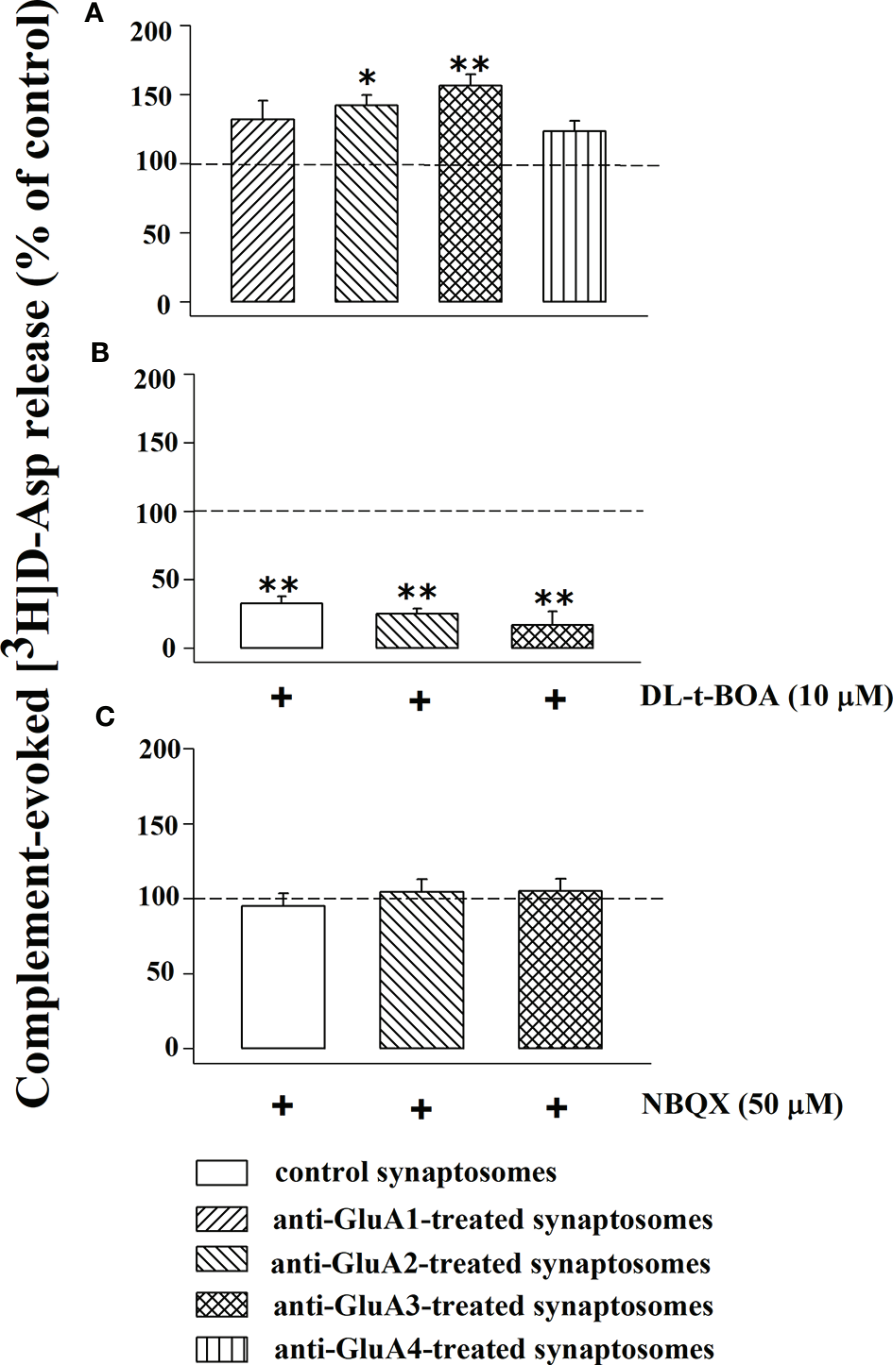
Complement evokes the [^3^H]D-aspartate release from mouse cortical synaptosomes incubated with anti-GluA receptors antibody: impact of DL-t-BOA and NBQX. Mouse cortical synaptosomes were incubated in the absence (control) or in the presence of anti-GluA1 (rising right hatched bar), anti-GluA2 (rising left hatched bar), anti-GluA3 (crossed hatched bar) and anti-GluA4 (vertical hatched bar) antibodies and the release of preloaded [^3^H]D-Asp was elicited by exposing them in superfusion to the complement (dilution 1:300) alone **(A)**, in the presence of 10 µM DL-t-BOA **(B)** and in the presence of 50 µM NBQX **(C)**. DL-t-BOA and NBQX were added concomitantly to complement. Results are expressed as percentage of the complement evoked-[^3^H]D-Asp release from untreated synaptosomes (% of control). The release of tritium evoked by complement (dilution 1:300) amounted to 2,69 ± 0.7 (% of the total synaptosomal tritium content). Results are expressed as the mean ± SEM from 8 to 10 experiments run in triplicate. *p < 0.05 versus control; **p < 0.01 versus control.

In recent years we demonstrated that the transient exposure of synaptosomes to complement elicits the release of glutamate from synaptosomes isolated from different regions of the CNS, including the cortex, through a carrier-mediated mechanism, as suggested by the finding that the releasing activity was totally prevented by the excitatory aminoacid transporter blocker DL-t-BOA (47). Synaptosomes preloaded with [^3^H]D-Asp were incubated with the anti-GluA antibodies and then exposed in superfusion to the mouse complement (dilution 1:300) and the releasing activity elicited by the immune complex was compared to that observed in control synaptosomes. The results reported in **Figure 7A** unveil a significant enhancement of the complement-evoked releasing activity in synaptosomes incubated with the anti-GluA2 and the anti-GluA3 antibodies. Differently, incubation with the anti-GluA1 and the anti-GluA4 antibodies failed to affect the complement-evoked glutamate outflow. The Figure also shows that the concomitant presence of DL-t-BOA largely prevented the complement-evoked release of tritium from cortical synaptosomes incubated with the anti-GluA2 or the anti-GluA3 antibody to a level largely comparable to the reduction observed in untreated synaptosomes (**Figure 7B**). Differently, NBQX (50 µM) failed to affect the complement-evoked release of [^3^H]D-Asp from control and from anti-GluA2 and anti-GluA3 treated synaptosomes (**Figure 7C**), suggesting that AMPA receptors-mediated events do not participate to the complement-induced release from cortical glutamatergic synaptosomes.

### The Anti-GluA Antibody Induced Potentiation of the Complement-Evoked Release Depends on the C1q Component

Complement-mediated responses mainly rely on three different pathways, namely the classic, the alternative, and the lectin-dependent pathways. Among them, the classic pathway is preferentially triggered by the presence of antibody-antigen complexes and its activation involves the C1q component of the immunocomplex.

**Figure 8:**
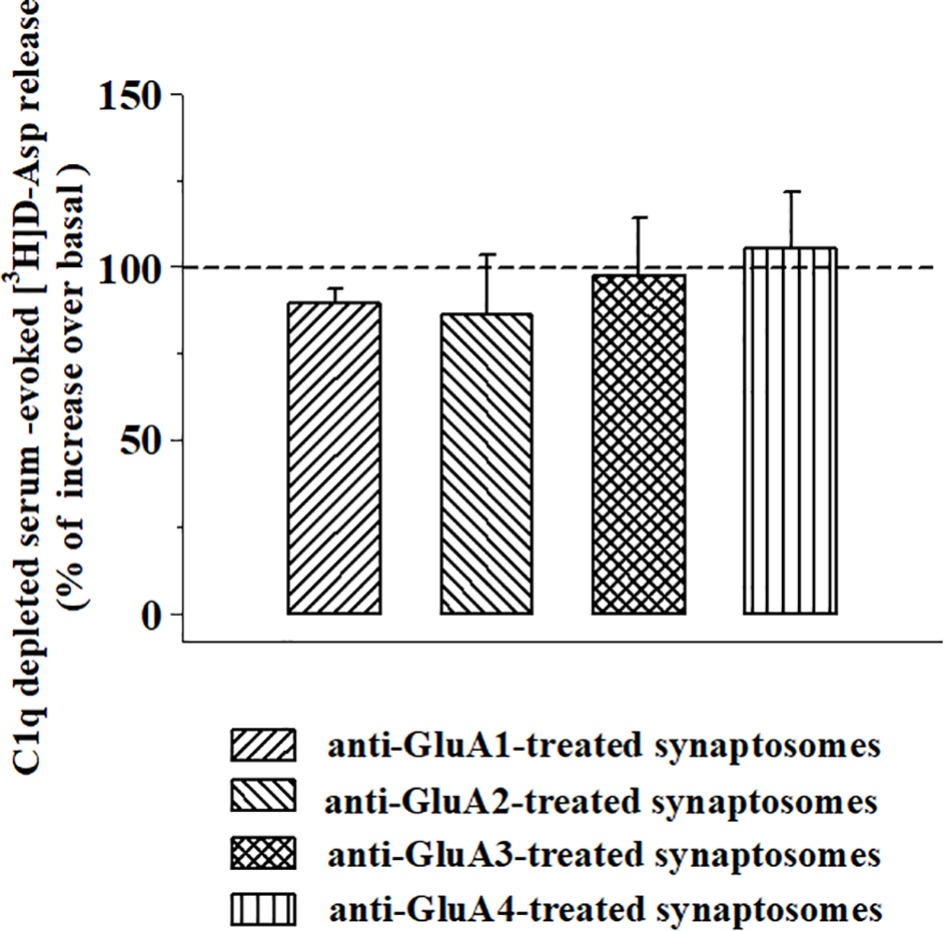
Effects of C1q-depleted complement on the [^3^H]D-aspartate release from mouse cortical synaptosomes incubated with anti-GluA receptors antibody. Mouse cortical synaptosomes were incubated in the absence (control) or in the presence of anti-GluA1 (rising right hatched bar), anti-GluA2 (rising left hatched bar), anti-GluA3 (crossed hatched bar), and anti-GluA4 (vertical hatched bar) antibodies, and the release of preloaded [^3^H]D-Asp was elicited in superfusion by exposing synaptosomes to the C1q-depleted complement (dilution 1:300). Results are expressed as percentage of increase of the C1q-depleted complement evoked-[^3^H]D-Asp release (% of control) and represent the mean ± SEM of 4 experiments in triplicate.

We asked whether the presence of the anti-GluA/GluA subunit protein complex at the aminoterminal of the synaptosomal plasma membranes could amplify the complement-induced releasing activity through the C1q-dependent pathway. To answer the question, synaptosomes incubated with the anti-GluA2 or the anti-GluA3 antibodies were exposed to C1q-depleted complement (1:300) in superfusion. The omission of the C1q component nulled the amplification of the releasing activity observed in the anti-GluA2 and the anti-GluA3 incubated synaptosomes (**Figure 8**) when compared to control synaptosomes.

## Discussion

It is known that the GluA subunit composition of the AMPA receptors is almost unpredictable, due to the lack of selective ligands for each subunit. To bypass the caveat, we applied the “immunopharmacological approach” (23, 26), which implies the use of commercially available anti-GluA antibodies as selective ligands to evidentiate the participation of selected subunits in the receptor assembly. By using synaptosomes isolated from the cortex of mice we investigated the impact of commercial antibodies recognizing the GluA1 to 4 subunits on the presynaptic AMPA autoreceptor controlling glutamate release to deciphering, if possible, their subunit composition. At the meantime, we hypothesized that the results from this study would highlight antibody-mediated changes to the (S)AMPA-induced releasing effects, improving the knowledge of the pathological events subserving autoimmune diseases typified by the production of auto-anti-GluA antibodies (19, 48–54)..

As far as the identification of the subunit composition is concerned, the approach was successful since the antibodies recognizing the different GluA proteins caused functional changes to the (S)AMPA-evoked releasing activity that we propose could correlate with the composition in GluA subunits. Well in line with the data in the literature (19, 49, 53, 54) the commercial anti-GluA antibodies amplified the releasing activity elicited by AMPA receptors in cortical glutamatergic nerve endings. The impact of the antibodies was strictly subunit-dependent, since it was observed only in synaptosomes treated with the anti-GluA2 and the anti-GluA3 antibodies, while the anti-GluA1 and anti-GluA4 ones were inactive. The anti-GluA2 and 3 antibodies amplified to a comparable extent the (S)AMPA-evoked glutamate release but did not cause evident changes to the spontaneous outflow of the transmitter, suggesting that they do not act as orthosteric ligands. Furthermore, the presence of saturating cyclothiazide (43) allows to exclude the possibility that the anti-GluA2 and 3 antibodies allosterically facilitate the interaction of the agonist with its binding site. Finally, NBQX inhibited the (S)AMPA-evoked release of tritium in both untreated and anti-GluA2/3 treated synaptosomes ruling out the possible involvement of non-specific event(s).

In an attempt to propose a molecular mechanism(s) accounting for the antibody-induced amplification of the (S)AMPA-evoked exocytosis, we proposed that the presence of the anti-GluA/GluA complex could have affected the insertion of the AMPA receptors in synaptosomal plasma membranes, as already observed for the NMDA receptors (26). The hypothesis deserves attention since AMPA receptors undergo both a constitutive and a regulated trafficking which dynamically control their signaling at chemical synapsis (44, 45, 55). The regulated trafficking occurs upon induction of synaptic plasticity, leading to long-lasting postsynaptic modifications. Differently, the constitutive trafficking develops in few minutes, independently on the synaptic activity and it occurs also presynaptically (30, 39, 40) where it mainly involves AMPA receptors composed of GluA2/GluA3 subunits (44). Our results clearly indicate that the AMPA autoreceptors in cortical synaptosomes traffic in a constitutive manner, since hindering the GluA2/GRIP1/PICK1 with pep2-SVKI amplifies the (S)AMPA-mediated releasing activity. The involvement of altered in-out movements of the ionotropic receptor relies on (i) the dynamic feature of the receptor under study and (ii) the results from biotinylation studies demonstrating that both the anti-GluA2 and anti-GluA3 antibodies elicited comparable modifications to the insertion of the GluA2 and the GluA3 subunits in synaptosomal plasma membranes. Notably, a recent study demonstrated in cortical specimens from patients suffering from frontotemporal lobar degeneration associated to an increase availability of anti-GluA3 auto-antibodies the onset of postsynaptic changes in the expression of scaffolding proteins (e.g., the GRIP1/PICK1 ratio) involved in AMPA receptor retention/internalization (27). The possibility that this adaptation might represent the consequence of impaired in-out movements of the GluA2 and 3 subunits due to the circulating anti-GluA autoantibodies and that it also could occur at the presynaptic level deserves attention and will be tackled in future studies.

In general, the two antibodies increased, although to a different extent, the density of both the subunits. However, while the density of the GluA2 subunits was slightly but significantly increased by both antibodies, the density of the GluA3 subunits was hugely boosted, almost doubling the protein content with respect to controls. These results contrast the findings by Peng and colleagues (19) showing that a commercially available anti-GluA2 antibody was *per se* unable to affect AMPA receptor internalization in cultured hippocampal neurons, but significantly modified it when a secondary antibody was added to cross-linking the primary antibody. Differences in the tissue preparations (cortical synaptosomes versus cultured hippocampal neurons) as well as in the experimental approaches (the timing of the exposure and the dilution of the antibody) might account for the different results.

The main consequence of the incubation of the synaptosomes with both the antibodies is that the GluA2:GluA3 ratio largely decreased in the treated synaptosomes due to the huge increase of the GluA3 component. This would impact severely the functional properties of the AMPA autoreceptors, particularly affecting the ions conductance of the associated channel. Yet, while the edited GluA2(R) (56), the one that prevails in the brain of mammals (57, 58), makes the AMPA receptors largely Ca^2+^ impermeable (55, 59, 60), the GluA3 subunits improve its calcium permeability (61, 62). The reduced GluA2: GluA3 ratio in the antibody -treated synaptosomes would favor the Ca^2+^ influx through the AMPA receptor-associated channels, then amplifying glutamate exocytosis, as indeed observed in release experiments. This outcome would impact the synaptic plasticity and therefore the neurological performances in patients with circulating anti-GluA3 autoantibodies (21, 53, 63), including those suffering from Rasmussen’s encephalitis (RE) (64).

Several studies were dedicated to deciphering the mechanisms accounting for the neurological deterioration that develop in patients suffering from RE and, as far as the role of the of the anti-GluA3 antibodies is concerned, two main cascades of events were proposed to account for the central excitotoxicity that typify the course of the disease.

The first one involves an excessive activation of the AMPA autoreceptors elicited by anti-GluA3 antibodies, as indeed here observed with the commercial antibody, that could mediate excitotoxicity in neurons.

The second one implicates an anti-GluA3-induced complement-mediated pathway [discussed by (20), but see also (53)]. While studying the impact of complement in synaptosomes, particularly on the release of glutamate, we demonstrated that the immune complex releases this transmitter from nerve endings isolated from different CNS regions, including the cortex and that its releasing activity is amplified by the presence of antibody-antigen complexes at the outer side of synaptosomal membranes (64). Accordingly, the results described in this works unveil that (i) the complement-evoked glutamate release is significantly potentiated in synaptosomes treated with the anti-GluA2 and 3 antibodies, (ii) the reinforcement of the releasing activity strictly depends on the C1q-mediated classic pathway of activation of the immunocomplex (65, 66); (iii) the complement-evoked releasing activity in both anti-GluA treated and untreated synaptosomes did not involve AMPA-mediated events, but carrier-mediated processes.

Before drawing any conclusions, it is worth reminding the significant reduction, instead of an amplification, of the (S)AMPA-evoked release of glutamate described in the recent study from Palese and colleagues (19). Several events might account for these observations that apparently contrast the present results. First, the possibility that the commercial anti-GluA3 antibody and the autoantibodies in the patient’s serum target different sequences within the NH2 terminus of the receptor subunits (52, 54). Second, the fact that the serum could contain endogenous components that compensate for the antibody-induced adaptation of the AMPA autoreceptors (19). The data so far available does not allow to draw a definitive conclusion and further studies are required to correctly address the question.

To conclude, beside allowing the prediction of the subunit composition of the presynaptic release-regulating AMPA receptors controlling glutamate exocytosis, the results of this study describe *complement-independent* and *complement-dependent* events triggered by anti-GluA antibodies in cortical glutamatergic nerve endings that cause excessive glutamate release and that might be relevant to the progression of synaptic derangements and excitotoxicity in autoimmune diseases (53). The *complement-independent* pathway relies on a cascade of events involving AMPA autoreceptors and implies changes in the constitutive in-out movements of the GluA2 and 3 subunits in plasma membranes leading to an increased insertion of AMPA receptors. The *complement-dependent* pathway depends on the activation of the complement trough the classic pathway and it is triggered by the presence of the antibody-antigen complex in synaptosomal membranes. Taking into consideration that the production anti-GluA antibodies is observed in several autoimmune pathologies including RE, limbic encephalitis, epilepsy, sporadic olivopontocerebellar atrophy, and multiple sclerosis (19, 21, 49, 53, 67, 68), but also in patients following antibody therapy (69) and bone marrow transplantation (70), our observations could help to decipher some of the pathological events that typify disease progression, but also underlie adverse effects during disease therapies.

## Data Availability Statement

The original contributions presented in the study are included in the article. Further inquiries can be directed to the corresponding author.

## Ethics Statement

The animal study was reviewed and approved by Local Committee for Animal Care and Welfare, University of Genova and Italian Ministry of Health.

## Author Contributions 

AP designed the experiments, supervised the execution of the research activity and the statistical analysis, and wrote the manuscript. FC and GO performed release experiments and western blot analysis. CU performed confocal microscopy analysis. SM, GV, and SM-F supported the scientific data analysis and discussion and revised the manuscript. FC, GO, CU, GV, SM, SM-F and AP approved the final version of the manuscript and agree to be accountable for all the aspects of the work. All authors contributed to the article and approved the submitted version.

## Conflict of Interest

The authors declare that the research was conducted in the absence of any commercial or financial relationships that could be construed as a potential conflict of interest.

## Abbreviations

[^3^H]D-Asp, [^3^H]D-aspartate; CNS, central nervous system; VGLUT1, vesicular glutamate transporter type 1; CCR, C-C Chemokine Receptor; GluA, glutamate AMPA; RE, Rasmussen’s encephalitis; NBQX, 2,3-Dioxo-6-nitro-1,2,3,4-tetrahydrobenzo[f]quinoxaline-7-sulfonamide disodium salt; DL-t-BOA, DL-threo-β-Benzyloxyaspartic acid.
